# Periostin in Angiogenesis and Inflammation in CRC—A Preliminary Observational Study

**DOI:** 10.3390/medicina58010096

**Published:** 2022-01-08

**Authors:** Agnieszka Kula, Miriam Dawidowicz, Sylwia Mielcarska, Paweł Kiczmer, Magdalena Chrabańska, Magdalena Rynkiewicz, Elżbieta Świętochowska, Dariusz Waniczek

**Affiliations:** 1 Department of Oncological Surgery, Faculty of Medical Sciences in Zabrze, University of Silesia, 35 Ceglana, 40-514 Katowice, Poland; miriamdawidowicz@o2.pl (M.D.); dariusz.waniczek@gmail.com (D.W.); 2 Department of Medical and Molecular Biology, Faculty of Medical Sciences in Zabrze, Medical University of Silesia, 19 Jordana, 41-800 Zabrze, Poland; sylwiamielcarska@gmail.com (S.M.); elaswieta@interia.pl (E.Ś.); 3 Department and Chair of Pathomorphology, Faculty of Medical Sciences in Zabrze, Medical University of Silesia, 13-15 3 Maja, 41-800 Zabrze, Poland; Pawel.kiczmer@protonmail.com (P.K.); hannaonyszczuk@sum.edu.pl (M.C.); mrynkiewicz@sum.edu.pl (M.R.)

**Keywords:** periostin, colorectal cancer, tumor microenvironment

## Abstract

*Background and Objectives*: To assess the periostin level and the concentrations of pro-inflammatory cytokines: TNFα, IFN-γ, IL-1β and IL-17 in tumor and marginal tissues of CRC and to investigate the influence of periostin on angiogenesis by MVD (microvessel density) and concentration of VEGF-A in relation to clinicopathological parameters of patients. *Materials and Methods*: The study used 47 samples of tumor and margin tissues derived from CRC patients. To determinate the concentration of periostin, VEGF-A, TNFα, IFNγ, IL-1β and IL-17, we used the commercially available enzyme- linked immunosorbent assay kit. MVD was assessed on CD34-stained specimens. The MVD and budding were assessed using a light microscope *Results:* We found significantly higher concentrations of periostin, VEGF-A, IFN-γ, IL-1 β, IL-17 and TNFα in the tumor samples compared with surgical tissue margins. The tumor concentrations of periostin were correlated with tumor levels of VEGF-A, IFN-γ, IL-1β and TNFα. We observed significant correlation between margin periostin and VEGF-A, IFN-γ, IL-17 and TNFα in tumor and margin specimens. Additionally, we found a significantly negative correlation between periostin tumor concentration and microvessel density at the invasive front. Tumor periostin levels were also correlated positively with tumor budding. *Conclusions:* Periostin activity may be associated with pro-inflammatory cytokine levels: TNFα, IFN-γ, IL-1β and IL-17. Our results also suggest the role of periostin in angiogenesis in CRC and its upregulation in poorly vascularized tumors. Further research on the regulations between periostin and cytokines are necessary to understand the interactions between tumor and immune tumor microenvironment, which could be helpful in the development of new targeted therapy.

## 1. Introduction

Colorectal cancer (CRC) is the second and third most common cancer in females and males, respectively, accompanied by high rates of morbidity and mortality. According to Global Cancer Statistics, the prevalence of CRC in 2018 was about 6.1% of all new cancer cases [1]. A 60% increase in CRC cases is expected worldwide by 2030 [2].

Several risk factors are associated with the incidence of this malignancy, including obesity, red meat intake, cigarette smoking, alcohol intake, low physical activity and low vegetable consumption [3,4]. CRC may also be caused by genetic instabilities, including chromosomal instability, microsatellite instability (MSI) and CpG islands methylator phenotypes [5].

Periostin is a multifunctional, extracellular matrix protein, which shares structural homology with the insect cell adhesion molecule FASCICLIN 1, and functions as a ligand for some integrins. The binding of periostin to integrins initiates a cross-talk between the integrins and receptor tyrosine kinases such as EGF at the plasma membrane. These interacting molecules co-activate the serine threonine Akt and Erk cell signaling pathway, which modulates cell motility, proliferation and survival. Periostin is rarely detected in most normal adult tissues, but is highly induced in lesions, inflammation, and several forms of cancers, including pancreatic, ovarian, lung, breast, gastric, esophageal and colon [6,7,8].

Some reports have indicated that periostin plays an important role in numerous cancers. By binding to integrin avβ3 or avβ5 receptors, it can promote cell motility, cell survival and also adhesion, which is crucial in the tumor growth, angiogenesis, and metastasis [9].

Thus far, there have been several reports on the role of periostin in colorectal cancer. Ouyang G. et al. suggested that periostin potentially promotes metastatic growth of CRC by augmenting cell survival via the Akt/PKB pathway [10]. Wu G. et al. found periostin to be related to the liver metastasis of CRC and may be a potential target for CRC [11]. Ben Q-W et al. stated that levels of this protein may be of clinical value in identifying patients who may have aggressive form and metastasis of CRC [12].

CRC represents a paradigm for the link between inflammation and oncogenesis. Inflammation is associated with the accumulation of various immune cells and inflammatory mediators, such as cytokines, chemokines and growth factors [13,14]. Tumors have the ability to regulate differentiation, progression and promotion by remodel stroma and establish microenvironment. This process is often accompanied by the release of pro-inflammatory factors, whereas chronic inflammation is involved in all three stages of tumor development- initiation, promotion and progression [15]. The levels of several pro-inflammatory cytokines are elevated in colorectal cancer [14,16]. Shimoyama Y. et al. suggested that periostin has an anti-tumor effect on colitis-induced colorectal cancer [17]. Elliott et al. proved that the expression of periostin is related to an inflammatory microenvironment and the presence of TNFα in human chronic wounds [18]. In retinal neovascular diseases, pro-inflammatory cytokines TNFα and IFN-γ attenuate the periostin expression [19]. A potential synergistic effect of TNFα and IL-17 in periostin-mediated collagen deposition has been described in liver fibrosis [20].

It has been reported that in tumor microenvironment, there are different types of cells involved in tumor support and progression. The most important of them are tumor-associated macrophages (TAMs), myeloid-derived suppressor cells (MDSCs) and cancer-associated fibroblasts (CAFs) CAFs are responsible for the production of periostin, which is the subject of our research. Inside the TME, complex communication pathways have been observed allowing the tumor and the TME to independently influence one another. Several pro-inflammatory cytokines are involved in the regulation of processes between the TME and tumor. We selected cytokines which predominantly activate the NF-κB (nuclear factor κB), i.e., TNFα, IL-1β and IL-17, to be included in our study. NF-κB is a key mediator of innate immune responses and may play an important role in inflammation-associated cancer progression. IFN-γ is required for human major histocompatibility complex (MHC) class I and II expression, and thus plays a key role in tumor immunogenicity. However, it is becoming increasingly clear that IFN-γ may exert some tumor supportive effects [21].

In some immunological contexts, IFN-γ favors the activation of alternative signaling pathways such as STAT3, which is associated with tumor progression. In addition, the FAS1 domains of periostin bind to several integrins on the cell surface, causing the activation of signaling pathways, such as NF-κB and STAT3. NF-κB and STAT3 constitute a tangle of signaling pathways common to our selected cytokines and periostins [22]. Moreover, periostin mediates intestinal inflammation through the activation of NF-κB signaling [23].

For that reason, we wanted to investigate if there was any association between the molecules studied.

The proangiogenic activity of periostin is reported in gliomas, breast cancer, pancreatis cancer and liver metastases in CRC; however, in primary colorectal cancer, it has been poorly investigated [6,16,17,18]. In breast cancer the endothelial tip cells deliver periostin, thus contributing to the development of the perivascular niche [21,24]. In pancreatis cancer, a high expression of periostin induces tubule formation, whereas periostin knockdown results in decreasing VEGF expression. Periostin has been shown to induce angiogenesis in renal cell carcinoma via upregulating the IKL/AKT/mTOR pathway [25]. Similarly, revascularization and the progression of pancreatic neuroendocrine tumors (PNETs) under extended vascular-endothelial growth factor A blockade are dependent on periostin [26]. We used MVD (microvessel density) assessments to investigate the influence of periostin on angiogenesis. Additionally, we assessed the levels of the known angiogenic marker VEGF-A and its correlation with periostin levels. Data from studies on gastric cancer cell lines provide evidence that periostin is a hypoxia response gene and mediates communication between cancer cells and hypoxic endothelial cells, in part, through VEGF signaling [25]. Additionally, in non-small-cell lung cancer, it has been observed that increased periostin expression is a response of cancer cells to hypoxic conditions [10]. Moreover, in the present study, we assessed the tumor budding, which is an important morphological reflection of the invasive activity which precedes metastasis, and the switch from cell proliferation to invasion in buds might be triggered by hypoxia [26,27,28,29].

In this study, we wanted to investigate the correlations between periostin, angiogenesis and the selected cytokines—TNFα, IFN-γ, IL-1β and IL-17—in the context of TUMOR–MARGIN interactions in colorectal cancer. Levels of periostin, VEGF-A TNFα, IFN-γ, IL-1β and IL-17 were measured using ELISA tests, MVD was assessed on CD34-stained specimens, tumor budding was evaluated on H + E slides, and these data were compared with clinicopathological features of the patients.

## 2. Materials and Methods

The samples from 47 patients obtained during surgery due to CRC were used in the study. Patients were treated in the 1st Specialistic Hospital in Bytom, Poland (approval of the Research Ethics CommitteePCN/0022/KB1/42/VI/14/16/18/19/20). The collected specimens included colorectal tumor tissues and surgical tissue margins. Patients were enrolled in the study after meeting the following criteria: age > 18 years, signed written consent and histological confirmation of colorectal adenocarcinoma and surgical “tumor-free” tissue margin. The exclusion criteria were as follows: no consent to participate in the study, tumors other than adenocarcinoma, tumors with involved margins and age < 18 years. To classify the cancer stage, the TNM staging system and grading were used. Research sample characteristics are presented in Table 1.

### 2.1. Preparation of Samples for the Evaluation of Periostin, VEGF-A, TNFα, IFN-γ, IL-1β and IL-17

Fragments of the tumor tissue and surgical tissue margin were weighted and homogenized using a PRO 200 homogenizer (PRO Scientific Inc., Oxford, CT, USA) at 10,000 rpm in nine volumes of phosphate-buffered saline (BIOMED, Lublin, 06, Poland). The suspensions were sonicated with an ultrasonic cell disrupter (UP 100H, Hielscher Ultrasonics GmbH, Teltow, BB, Germany). Subsequently, the homogenates were centrifuged at 12,000 rpm for 5 min at 4 °C. The total protein level was determined using a Universal Microplate Spectrophotometer (μQUANT, Biotek Inc., Winooski, VT, USA).

### 2.2. Evaluation of Periostin, VEGF-A, TNFα, IFN-γ, IL-1β and IL-17

To assess the levels of the investigated proteins, an enzyme-linked immunosorbent assay (ELISA) was used, following to the manufacturer’s instructions. Periostin levels were evaluated by a human Periostin ELISA kit (Sigma Aldrich, Burlington, MA, USA) with a sensitivity of 80 pg/mL. Levels of VEGF-A, TNFα, IL-1β, IL-17 and IFN-γ were measured with ELISA Kits: Human VEGF-A ELISA KIT (Diaclone, Besancon Cedex, France, sensitivity of 7.9 pg/mL), Human TNFα ELISA KIT (Diaclone, Besancon Cedex, France, sensitivity of 10 pg/mL), Human IL-1β Elisa Kit (Biovendor, Brno, Czech Republic sensitivity of 0.4 pg/mL), Human IL-17a Elisa Kit (Diaclone, Besancon Cedex, France, sensitivity of 2.3 pg/mL), human IFN-γ ELISA kit (KHC4022 Invitrogen Waltham, MA, USA, sensitivity of 4 pg/mL). The absorbance of the samples was determined using a Universal Microplate Spectrophotometer (μQUANT, Biotek Inc., Winooski, VT, USA). The measurement was conducted at a wavelength of 450 nm. The obtained results were recalculated to the corresponding total protein level and presented as g/g of protein.

### 2.3. Immunostaining

CD 34 immunostaining was performed in 24 randomly selected cases. Tissue samples were obtained from formalin-fixed paraffin-embedded tissue blocks with primary CRC and tumor-free margin samples. Then, the samples were deparaffinized and rehydrated. We then performed antigen recovery by boiling the slides in EnVision Flex Target Retrieval Solution High pH (Dako, Carpinteria, CA, USA) for 20 min at 95 °C. Prepared samples were incubated with peroxidase blocked reagent (Dako, Carpinteria, CA, USA) and then incubated with CD34 antibody (clone: QBEnd/10 Cell Marque, Rocklin, CA, USA; incubation time: 30′; dilution: 1:150). In the next step, the samples were placed in EnVision FLEX HRP (Dako, Carpinteria, CA, USA). The antigen-antibody complexes were then stained with 3,3′-diaminobenzidine. Finally, tissue sections were counterstained with hematoxylin, dehydrated and covered with coverslips for further analysis.

### 2.4. Histological Evaluation

Histological evaluation was performed by two independent pathologists using an Olympus BX51 microscope. MVD was assessed on CD34-stained specimens by two independent pathologists in the regions of the invasive tumor front, counting the largest number of microvessels per area. Tumor sections were initially assessed under low magnification to detect an invasive tumor front; then, three high vascular hotspots were selected. Microvessels were counted in three fields of view under magnification X20. MVD is presented as the mean number of microvessels in the assessed fields of view; the number has been adjusted by the normalization factor (1.210). Tumor budding was assessed in the same 30 samples. Tumor buds were assessed in one field of view (FOV) in the hotspot area at the invasive front at X20 magnification. The number of buds was adjusted with the normalization factor (1.210). Budding is noted as proposed: low budding: 0–4 buds; intermediate budding: 5–9 buds; high budding: >10 buds. The mean number of buds per FOV was also used in the statistical analysis.

### 2.5. Statistical Analysis

Data distribution was assessed using the Shapiro–Wilk test. The log transformation of the levels of the examined molecules provided a better fit to the normal distribution. Data are presented as mean ± SD (variables with normal distribution). To compare the tumor and margin levels, paired Student’s *t*-tests were performed. Independent variables were also compared using Student’s *t*-tests. The Pearson’s coefficient was used to assess the relationships between variables with normal distribution. For those with non-normal distributions Spearman’s correlation coefficient was used. *p* values < 0.05 were considered statistically significant. The statistical analysis was performed using STATISTICA 13 software (Statsoft Tulsa, OK, USA).

## 3. Results

### 3.1. Results from Tissue Homogenates

**Figure 1:**
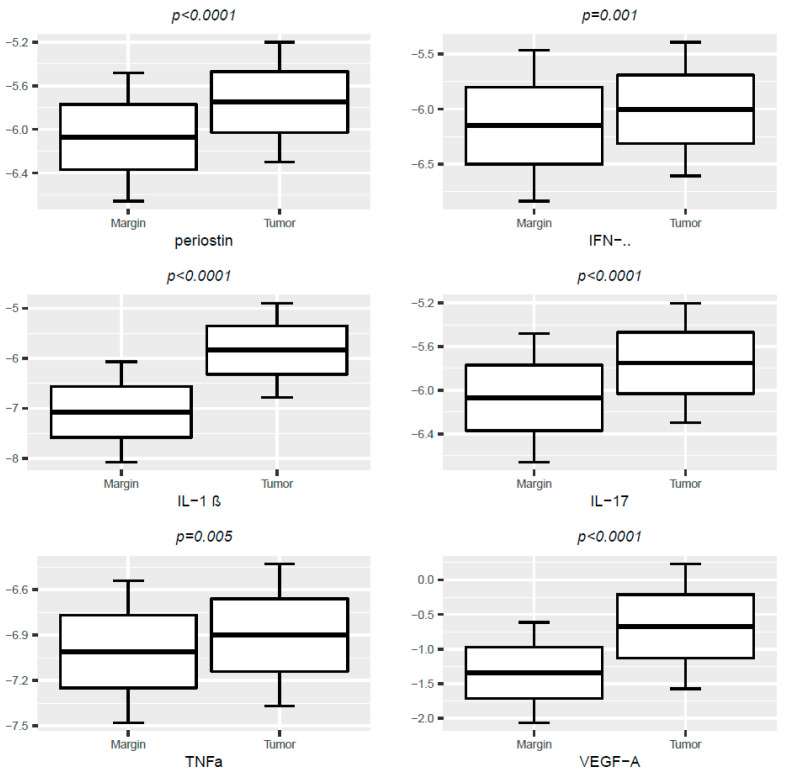
Box-plot- levels of periostin, VEGF-A, IFN-γ, IL-1 β, IL-17, TNFα molecules in tumor and tissue margins; protein levels are presented as log-transformed values as g/g. Paired T-student’s test.

We found significantly higher concentrations of periostin, VEGF-A, IFN-γ, IL-1 β, IL-17 and TNFα in the tumor samples compared with surgical tissue margins (Table 2, Figure 1).

**Figure 2:**
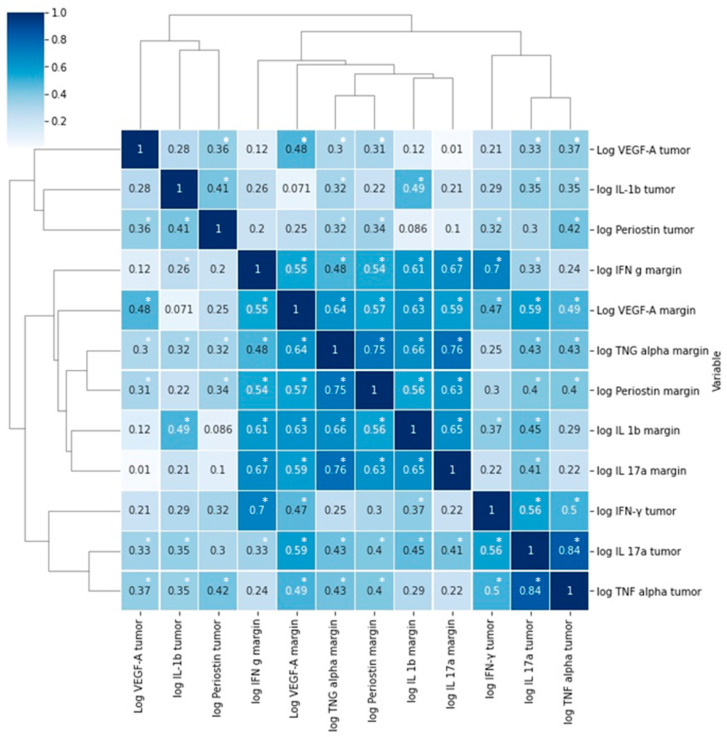
Correlations between the levels of the examined molecules presented as a heatmap with hierarchic clustering. R—Pearson’s correlation coefficient, * *p* < 0.05.

We found significant correlations between periostin levels in tumor and VEGF-A, IFN-γ, IL-1β, and TNFα concentrations in tumor (Figure 1, Table 3). A significant correlation was found between periostin levels in margin and VEGF-A, IFN-γ IL-1 β, IL-17, and TNFα in tumor and margin specimens (Figure 2, Table 3).

### 3.2. Association with Histologic Features

**Figure 3:**
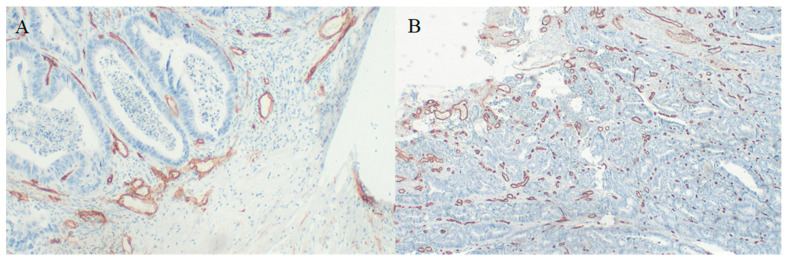
CD34 immunostaining in CRC specimens used to assess microvessel density (**A**) 400× magnification, (**B**) 100× magnification.

**Figure 4:**
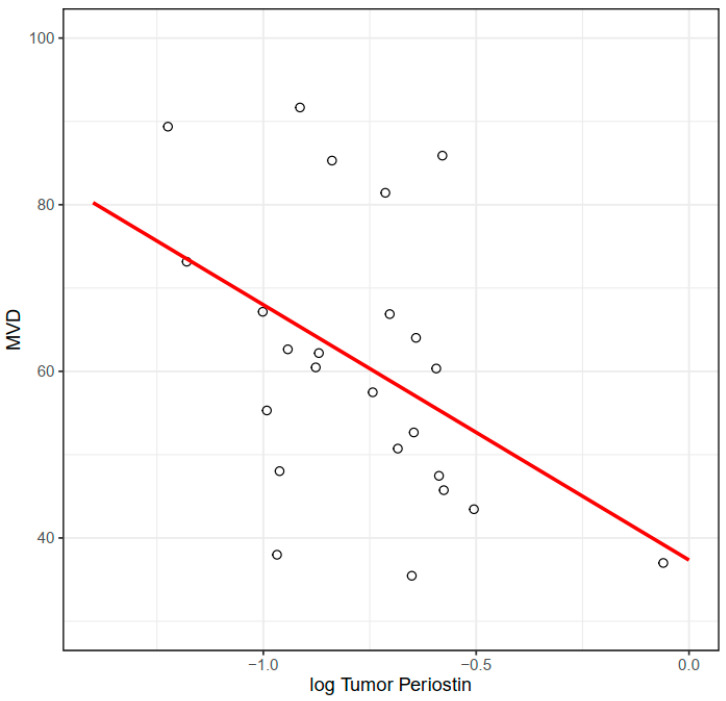
Association between MVD at the invasive front and periostin concentration (R = −0.44, *p* = 0.036). R—Pearson’s correlation coefficient.

**Figure 5:**
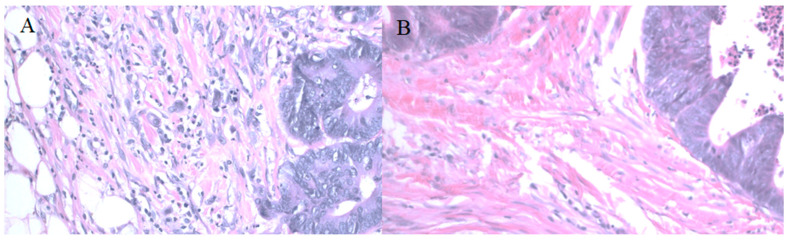
Budding assessment (200× magnification, Olympus BX 51 microscope, Olympus Life Science, Olympus manufacturer, Tokyo, Japan), (**A**) high budding tumor (6 buds in the camera field of view (FOV), more than 10 buds in full microscope FOV), (**B**) Low budding tumor, 1 bud in FOV.

For 30 cases we obtained HE-stained specimens; additionally, for 24 cases, we obtained paraffin-embedded specimens from tumors, which allowed us to perform CD34 immunostaining to assess microvessel density (Figure 3). We found a significant negative correlation between periostin tumor concentration and microvessel density at the invasive front (Figure 4). There were no significant correlations between MVD and other molecules. Tumor periostin was also correlated positively with tumor budding (Table 4, Figure 5).

We did not find any significant correlation between the investigated molecules and clinical features such as TNM scale, stage and grading.

## 4. Discussion

### 4.1. The Role of Periostin in Tumorigenesis

Periostin is a matricellular protein in tissue microenvironment and is often associated with the extracellular matrix (ECM)**.** The ECM is a crucial component of the tissue microenvironment, and its proteins can modulate and regulate the behavior of surrounding cells and the homeostasis of tissues [30]. Periostin has the ability to bind integrins which initiate cross-talk between the integrins and receptor tyrosine kinases such as EGF at the plasma membrane. These connections lead to the activation of the serine threonine Akt and Erk cell signaling pathway, which modulates cell motility, proliferation and survival [17].

The process of tumor formation and progression is influenced by two factors, namely, genetic/epigenetic changes in the tumor cells and rearrangement of the components of the tumor microenvironment (TME) [31]. The TME primarily consists of the extracellular matrix, blood and lymphatic vessel networks, immune cells including tumor-associated macrophages (TAMs), myeloid-derived suppressor cells (MDSCs) and cancer-associated fibroblasts (CAFs). Cancer-associated fibroblasts neither revert to a normal phenotype nor undergo apoptosis. These cells have been implicated in the stimulation of the epithelial–mesenchymal transition (EMT), which is extremely important for cancer development [32]. Studies have reported that periostin, in addition to being produced by fibroblasts in a healthy tissue environment, is also produced by CAFs in the tumor microenvironment; therefore, it is involved in tumor development [33].

There are several mechanisms by which periostin influences tumorigenesis.

Firstly, studies suggest that periostin can facilitate the interactions between cancer cells and the tumor niche by connections with integrins to promote cell migration. The second crucial role of periostin in tumor development is the promotion of angiogenesis through the up-regulation of vascular endothelial growth factor receptor 2 expression [30]. Additionally, periostin can promote EMT in several types of cancer cells, contributing to the promotion of metastasis and invasion of cancer [34]. Finally, it has been reported that activating protein tyrosine kinase 7 periostin promotes cancer stemness [33].

### 4.2. Levels of Periostin and Its Correlation with MVD and Budding

In our study, we found significantly higher concentrations of periostin levels in cancer tissues than in tissue margins (*p* < 0.0001). Our findings are consistent with the results achieved by other researchers [35,36,37,38]. The meta-analysis conducted by Yang et al. confirmed higher levels of periostin in multiple solid tumors, including colorectal cancer [33]. Moreover, higher periostin levels were correlated with a number of clinicopathological parameters, such as lymphatic metastasis, vascular invasion and tumor differentiation. There was also a correlation of aberrantly high periostin levels with poor overall survival and with worse disease-free survival [37,38].

Additionally, we found a significant negative correlation between periostin concentration and MVD at the invasive front. Higher microvessel density was found to be associated with high periostin expression in prostatic cancer [39]. The expression of periostin in pericytes was associated with angiogenesis in central nervous system tumors [22,40]. Although our observation of negative correlations of the MVD with periostin level is in contrast to the results of this correlation in prostatic cancer, we hypothesize that in CRC, higher level of periostin is stimulated by hypoxia. This seems to be in line with our further findings of positive correlations between periostin tumor concentration and tumor budding. Budding is known to be associated with hypoxia and hypovascularization [41]. However, the study group was too small to definitely conclude the association between periostin level and MVD. Other research investigating periostin in relation to hypoxia seems to be in accordance with our findings [38]. We also found a positive correlation between the level of periostin and VEGF-A in tumors, as well as in marginal tissue homogenates. Vascular endothelial growth factor (VEGF) is one of the target genes transcriptionally activated by HIF-1. It is widely accepted that HIF-1α/VEGF signaling is essential for tumor angiogenesis [42,43]. The positive correlation between periostin and VEGF also suggests the cooperation of these two molecules in angiogenesis processes, especially in hypoxic environments. Periostin overexpression induced by hypoxia was observed in mouse models. Basing on literature data and our observations, we suspect that periostin is upregulated in poorly vascularized tissue which undergoes hypoxia [44].

### 4.3. Correlations between Levels of Periostin and Selected Cytokines: IFN-γ, IL-1β, TNFα and IL-17

Periostin is mainly produced and secreted to the ECM by fibroblasts. Under physiological conditions as a result of trauma and subsequent inflammation, fibroblasts are activated by pro-inflammatory cytokines to produce periostin, among others. This process was evaluated in wound healing models, in which activated fibroblasts proliferate and migrate into the wound site, produce ECM to restore damaged tissue, and interact with inflammatory, immune, and other cells to restore homeostasis [45]. Many features of fibroblast activation are shared in other contexts, including cancer, where activated CAFs can strongly influence the TME and are the primary source of periostin. CAFs have been established as a key component of the crosstalk between tumor cells and their microenvironment. These are heterogeneous populations of fibroblastic cells found in the microenvironment of solid tumors that play key roles as inflammatory mediators. One of the central mechanisms by which CAFs regulate tumor-promoting inflammation is by secreting cytokines and chemokines that recruit and modulate the function of innate and adaptive immune cells in the tumor microenvironment. These cells are involved in mediating tumor-promoting inflammation and affecting the tumor–immune system interaction. Pro-inflammatory signaling by CAFs is induced by paracrine signaling derived from initiated epithelia and/or resident immune cells, and can also be accelerated by autocrine signaling, e.g., TGF-β signaling result in activation of mammary CAFs [46,47]. In chronic inflammation, such as in liver fibrosis, pro-inflammatory cytokines including TNFα and IL-17 are elevated and continue to drive periostin production, and thus, fibrosis of the liver [20,48].

Chronic inflammation also occurs in colorectal cancer and the surrounding tissues. Tumor-infiltrating cells (TILs) at the beginning of tumorigenesis, along with other elements of the TME, provide a barrier with anticancer effects. As tumors develop, this barrier becomes disrupted and the TME landscape changes, promoting cancer progression. The existing inflammation around tumors provides a source of cytokines [32]. We found a significantly higher expression of IFN-γ in tumors than in the tissue margin (*p* = 0.001). Furthermore, we observed a correlation between the levels of IFN-γ and the TNFα level in tumor tissues (*p* < 0.0001). A similar correlation was found between these molecules in the marginal tissues (*p* = 0.001). IFN-γ is a known activator of macrophages which secrete TNFα [49]. The activated macrophages also produce another pro-inflammatory cytokine: IL-1β [50]. The levels of IL-1β in the tumor were highly elevated in the tumor tissues in comparison with adjacent normal tissues (*p* < 0.0001). We observed a significant correlation between the levels of IFN-γ and IL-1β in the tissue margin as well as correlations of IFN-γ and periostin in both tumors and tissue margins). The impact of IFN-γ on the periostin levels could be executed by three ways. Firstly, by direct effects on fibroblasts and CAFs through IFN-γR1 and IFN-γR2 receptors and possible activation of the alternative signaling pathways, such as STAT3 [49]. STAT3 is one of the known transcription factors upregulating the expression of periostin. Secondly, IFN-γ, through macrophages, could enhance the excretion of TNFα, and in turn, TNFα could induce the expression of periostin through another transcription factor: c-Jun [20]. Finally, IFN-γ enhances the production of not only TNFα, but also IL-1β from macrophages [51]. Nevertheless, this hypothesis needs carefully designed experiments to elucidate the exact impact of IL-1β and IFN-γ on the periostin levels. Despite the positive correlation found between IFN-γ and periostin, there is also some evidence that IFN-γ can attenuate the expression of periostin [19]; however, in a study where airway hyper-responsiveness and lung fibrosis were investigated, the neutralization of IFN-γ inhibited the combination of eosinophilic infiltration, lung fibrosis, periostin deposition and neutrophilic infiltration [52].

The role of interleukin-17 in colorectal cancer has been considered significant in previous studies. Il-17 can promote colorectal cancer tumorigenesis by many pathways and, according to scientists, it has potential to be a diagnostic marker and a new target in treatment of CRC. Several studies have shown that IL-17 has an important role in the metastasis and prognosis of CRC. In our research, the levels of IL-17 were higher in tumor tissue compared with surgical tissue margins (*p* < 0.0001).

IL-17 is a pro-inflammatory cytokine, and its main source is a subpopulation from CD+ T cells known as T-helper17 (Th17) cells. In our study, the levels of periostin in the tissue margin correlated positively with the margin and tumor levels of IL-17. In their research on liver fibrosis, Amara et al. found that IL-17 induced fibrogenesis through periostin. Interestingly, this study found a synergistic effect of IL-17 and TNFα in periostin promotion [20]. Hus I. et al. described that tumor-associated macrophages have a significant impact on the production of Th17 cells, which secrete IL-17. TNFα can also be directly secreted by tumor-associated macrophages and Th17 cells [53].

TNFα is a central pro-inflammatory cytokine which is secreted with a series of inflammatory factors and cytokines by tumor-associated macrophages in the tumor microenvironment [54].

In our study, the levels of periostin in the tissue margin correlated positively with the margin and tumor levels of TNFα Additionally, we observed correlation between periostin and TNFα in tumor tissue. The correlation between these molecules may indicate the participation of TNFα in the regulation of periostin levels in the tumor microenvironment. Elliot et al. proved that, in human chronic wounds, the expression of periostin is related to the presence of TNFα [13]. In a study on intestinal epithelial cells, tumor necrosis factor upregulated the expression of periostin, whereas in other studies, TNFα has been shown to stimulate fibroblast proliferation [23,55]. On the other hand, TNFα has been shown to attenuate the periostin expression in retinal Müller glia [19]; however, TNFα and IL-17 work synergistically to enhance the expression of periostin, e.g., in liver inflammatory injury, which eventually leads to fibrosis. Not just limited to the liver, the synergistic effect of TNFα and IL-17 on periostin expression has also been noted in other inflammatory diseases, such as psoriasis, in which it leads to altered keratinocyte differentiation [20,56,57].

### 4.4. Lack of Correlation between Investigated Molecules and Clinical Features such as TNM Scale, Stage and Grading

In our study, we did not find any significant correlations between the investigated molecules and clinical features such as TNM scale, stage and grading.

This result is different from previous studies, and it is probably related to the limits of the research. First of all, it was limited by the small number of samples. Secondly, the research group was characterized by little differentiation. Out of 47 patients, 46 had the G2-parameter in grading scale. Moreover, the group was also characterized by low differentiation in TNM scale. Based on data from the literature, periostin is increasingly recognized as the molecule associated with poor prognosis, aggression of the histological grade, lymph node metastasis, distant metastasis and TNM stage of CRC [11,12]. It supports the cancer-supportive niche, cancer stem cell renewal and perivascular niche with angiogenesis [11,34,40,58]. In contrast, periostin might have anti-tumor properties. The periostin knock-out mice exhibited increased colitis-induced colon cancer development. Moreover, in this study it also was shown that periostin can promote cancer cell apoptosis, both in vivo and in vitro, and thus contribute to inhibition of the tumor growth [17].

## 5. Conclusions

The immune tumor microenvironment is a complex network of tumor, immune cells, stromal cells and extracellular matrix [59]. The cross-talk between immune cells and stromal cells has been widely investigated, and is still not fully understood. The results of our study indicate that the interplay between cytokines and fibroblasts is multidirectional and needs further research to understand the processes underlying periostin expression. The role of periostin in multiple solid tumors is pleiotropic; therefore, it seems that exploring its regulation is crucial in cancer research [24]. Thus, investigating the role of periostin in CRC may contribute to developing better understanding of this malignancy and identifying new targets for the therapy of CRC. Although we did not find any correlation between periostin and clinicopathological features, this could have been a result of the characteristics of our study group [36,37,38]. Tissues used in our study came from patients undergoing surgical treatment with oligo-metastatic or no-metastatic CRC; therefore, the group was not very diverse. In adults, the level of periostin under physiological conditions is low. Periostin expression is associated with inflammation [18]. However, the exact communication pathways between cytokines and fibroblasts producing it and its influence on the modification of the extracellular matrix require further research. Nevertheless, it seems that periostin-targeted therapy in CRC could potentially be the right direction, because periostin plays multiple roles in tumor development, and is crucial in metastasis [40]. Moreover, our findings suggest the role of periostin in angiogenesis in CRC and its upregulation in poorly vascularized tumors. Due to the low expression of periostin in healthy tissues, therapy aimed at modulating periostin expression could have fewer side effects. Unraveling the complex network of connections between extracellular matrix proteins, such as periostin, and the inflammatory infiltrate in response to cancer, is crucial for the development of further therapeutic options.

## Figures and Tables

**Table 1:** Characteristics of the patients.

	Female	Male	
	19	28	47 (100%)
Age	63.13 ± 10.95	61.90 ± 9.14	62.43 ± 9.83
T parameter			
T1	0 (0%)	0 (0%)	0 (0%)
T2	7 (36.84%)	5 (17.86%)	12 (25.53%)
T3	9 (47.37%)	14 (50.00%)	23 (48.94%)
T4	3 (15.79%)	9 (32.14%)	12 (25.53%)
N parameter			
N0	9 (47.37%)	12 (42.86%)	21 (44.68%)
N1	8 (42.11%)	9 (32.14%)	17 (36.17%)
N2	2 (10.53%)	7 (25.00%)	9 (19.15%)
M parameter			
M0	17 (89.47%)	19 (40.43%)	36 (76.60%)
M1	2 (10.53%)	9 (32.14%)	11 (23.40%)
TNM stage			
I	6 (31.58%)	4 (14.29%)	10 (21.28%)
II	4 (21.05%)	7 (25.00%)	11 (23.40%)
III	7 (36.84%)	8 (28.57%)	15 (31.94%)
IV	2 (10.53%)	9 (32.14%)	11 (23.40%)
Grading			
G1	1 (5.26%)	0 (0%)	1 (2.13%)
G2	18 (94.74%)	28 (100%)	46 (97.87%)
G3	0 (0%)	0 (0%)	0 (0%)

**Table 2:** Levels of periostin, VEGF-A, IFN-γ, IL-1 β, IL-17 and TNFα molecules in tumor and tissue margins; protein levels are presented as log-transformed values as g/g. Paired Student’s *t*-test.

	Tumor		Margin		*p*
	Mean	SD	Mean	SD	
Periostin	−5.75	0.28	−6.07	0.30	<0.0001
IFN-γ	−6.00	0.31	−6.15	0.35	0.001
IL-1 β	−5.84	0.48	−7.07	0.51	<0.0001
IL-17	−6.75	0.28	−6.96	0.30	<0.0001
TNFα	−6.90	0.24	−7.01	0.24	0.005
VEGF-A	−0.67	0.46	−1.34	0.37	<0.0001

**Table 3:** Correlations between the periostin levels and the examined molecules. R—Pearson’s correlation coefficient.

Pair of Variables	R	*p*
Tumor log periostin and tumor log IFN-γ	0.35	0.032
Tumor log periostin and tumor log IL-1 beta	0.40	0.012
Tumor log periostin and tumor log TNFα	0.40	0.013
Tumor log periostin and tumor log VEGF-A	0.36	0.014
Margin log periostin and tumor log IFN-γ	0.35	0.030
Margin log periostin and margin log IFN-γ	0.57	<0.0001
Margin log periostin and margin log IL-1 beta	0.64	<0.0001
Margin log periostin and tumor log IL-17	0.39	0.015
Margin log periostin and margin log IL-17	0.62	<0.0001
Margin log periostin and tumor log TNFα	0.45	0.005
Margin log periostin and margin log TNFα	0.80	<0.0001
Margin log periostin and tumor log VEGF-A	0.31	0.037
Margin log periostin and margin log VEGF-A	0.57	<0.0001

**Table 4:** Correlation of the periostin concentration with budding.

Pair of Variables	Tau–Kendall’s Correlation	
	Tau	*p*
Tumor periostin and budding	0.30	0.02

Budding and MVD correlation was negative and marginally significant (R = −0.38, *p* = 0.06).

## References

[1] Keum N., Giovannucci E. (2019). Global burden of colorectal cancer: Emerging trends, risk factors and prevention strategies. Nat. Rev. Gastroenterol. Hepatol..

[2] Al-Zalabani A. (2020). Preventability of Colorectal Cancer in Saudi Arabia: Fraction of Cases Attributable to Modifiable Risk Factors in 2015–2040. Int. J. Environ. Res. Public Health.

[3] Johnson C.M., Wei C., Ensor J.E., Smolenski D.J., Amos C.I., Levin B., Berry D.A. (2013). Meta-analyses of colorectal cancer risk factors. Cancer Causes Control.

[4] Villanueva C.M., Espinosa A., Gracia-Lavedan E., Vlaanderen J., Vermeulen R., Molina A.J., Amiano P., Gómez-Acebo I., Castaño-Vinyals G., Vineis P. (2021). Exposure to widespread drinking water chemicals, blood inflammation markers, and colorectal cancer. Environ. Int..

[5] Yaghoubi N., Soltani A., Ghazvini K., Hassanian S.M., Hashemy S.I. (2019). PD-1/ PD-L1 blockade as a novel treatment for colorectal cancer. Biomed. Pharmacother..

[6] Gillan L., Matei D., Fishman D.A., Gerbin C.S., Karlan B.Y., Chang D.D. (2002). Periostin secreted by epithelial ovarian carcinoma is a ligand for alpha(V)beta(3) and alpha(V)beta(5) integrins and promotes cell motility. Cancer Res..

[7] Moniuszko T., Wincewicz A., Koda M., Domysławska I., Sulkowski S. (2016). Role of periostin in esophageal, gastric and colon cancer. Oncol. Lett..

[8] Kusunose N., Akamine T., Kobayashi Y., Yoshida S., Kimoto K., Yasukochi S., Matsunaga N., Koyanagi S., Ohdo S., Kubota T. (2018). Contribution of the clock gene DEC2 to VEGF mRNA upregulation by modulation of HIF1α protein levels in hypoxic MIO-M1 cells, a human cell line of retinal glial (Müller) cells. Jpn. J. Ophthalmol..

[9] Deng X., Ao S., Hou J., Li Z., Lei Y., Lyu G. (2019). Prognostic significance of periostin in colorectal cancer. Chin. J. Cancer Res..

[10] Ouyang G., Liu M., Ruan K., Song G., Mao Y., Bao S. (2009). Upregulated expression of periostin by hypoxia in non-small-cell lung cancer cells promotes cell survival via the Akt/PKB pathway. Cancer Lett..

[11] Wu G., Wang X., Zhang X. (2013). Clinical implications of periostin in the liver metastasis of colorectal cancer. Cancer Biother. Radiopharm..

[12] Ben Q.-W., Zhao Z., Ge S.-F., Zhou J., Yuan F., Yuan Y.-Z. (2009). Circulating levels of periostin may help identify patients with more aggressive colorectal cancer. Int. J. Oncol..

[13] Janakiram N.B., Rao C.V. (2014). The role of inflammation in colon cancer. Adv. Exp. Med. Biol..

[14] Gallo G., Vescio G., de Paola G., Sammarco G. (2021). Therapeutic Targets and Tumor Microenvironment in Colorectal Cancer. J. Clin. Med..

[15] Sueyama T., Kajiwara Y., Mochizuki S., Shimazaki H., Shinto E., Hase K., Ueno H. (2021). Periostin as a key molecule defining desmoplastic environment in colorectal cancer. Virchows Arch..

[16] Klampfer L. (2011). Cytokines, inflammation and colon cancer. Curr. Cancer Drug Targets.

[17] Shimoyama Y., Tamai K., Shibuya R., Nakamura M., Mochizuki M., Yamaguchi K., Kakuta Y., Kinouchi Y., Sato I., Kudo A. (2018). Periostin attenuates tumor growth by inducing apoptosis in colitis-related colorectal cancer. Oncotarget.

[18] Elliott C.G., Forbes T.L., Leask A., Hamilton D.W. (2015). Inflammatory microenvironment and tumor necrosis factor alpha as modulators of periostin and CCN2 expression in human non-healing skin wounds and dermal fibroblasts. Matrix Biol..

[19] Peng Y.-Q., Cao M.-J., Yoshida S., Zhang L.-S., Zeng H.-L., Zou J.-L., Kobayashi Y., Nakama T., Shi J.-M., Jia S.-B. (2019). Attenuation of periostin in retinal Müller glia by TNF-α and IFN-γ. Int. J. Ophthalmol..

[20] Amara S., Lopez K., Banan B., Brown S.-K., Whalen M., Myles E., Ivy M.T., Johnson T., Schey K.L., Tiriveedhi V. (2015). Synergistic effect of pro-inflammatory TNFα and IL-17 in periostin mediated collagen deposition: Potential role in liver fibrosis. Mol. Immunol..

[21] Ghajar C.M., Peinado H., Mori H., Matei I.R., Evason K.J., Brazier H., Almeida D., Koller A., Hajjar K.A., Stainier D.Y.R. (2013). The perivascular niche regulates breast tumour dormancy. Nat. Cell Biol..

[22] Bernardes S.S., Pinto M.C.X., Amorim J.H., de Carvalho Azevedo V.A., Resende R.R., Mintz A., Birbrair A. (2020). Glioma Pericytes Promote Angiogenesis by Producing Periostin. Cell. Mol. Neurobiol..

[23] Koh S.-J., Choi Y., Kim B.G., Lee K.L., Kim D.W., Kim J.H., Kim J.W., Kim J.S. (2016). Matricellular Protein Periostin Mediates Intestinal Inflammation through the Activation of Nuclear Factor κB Signaling. PLoS ONE.

[24] Liu Y., Huang Z., Cui D., Ouyang G. (2019). The Multiaspect Functions of Periostin in Tumor Progression. Adv. Exp. Med. Biol..

[25] Liu Y., Li F., Gao F., Xing L., Qin P., Liang X., Zhang J., Qiao X., Lin L., Zhao Q. (2016). Periostin promotes tumor angiogenesis in pancreatic cancer via Erk/VEGF signaling. Oncotarget.

[26] de Smedt L., Palmans S., Sagaert X. (2016). Tumour budding in colorectal cancer: What do we know and what can we do?. Virchows Arch..

[27] Lugli A., Zlobec I., Berger M.D., Kirsch R., Nagtegaal I.D. (2021). Tumour budding in solid cancers. Nat. Rev. Clin. Oncol..

[28] Hatzikirou H., Basanta D., Simon M., Schaller K., Deutsch A. (2012). ’Go or grow’: The key to the emergence of invasion in tumour progression?. Math. Med. Biol..

[29] Dawson H., Koelzer V.H., Karamitopoulou E., Economou M., Hammer C., Muller D.-E., Lugli A., Zlobec I. (2014). The apoptotic and proliferation rate of tumour budding cells in colorectal cancer outlines a heterogeneous population of cells with various impacts on clinical outcome. Histopathology.

[30] Liu A.Y., Zheng H., Ouyang G. (2014). Periostin, a multifunctional matricellular protein in inflammatory and tumor microenvironments. Matrix Biol..

[31] Esfahani K., Roudaia L., Buhlaiga N., Del Rincon S.V., Papneja N., Miller W.H. (2020). A review of cancer immunotherapy: From the past, to the present, to the future. Curr. Oncol..

[32] Kosmidis C., Sapalidis K., Koletsa T., Kosmidou M., Efthimiadis C., Anthimidis G., Varsamis N., Michalopoulos N., Koulouris C., Atmatzidis S. (2018). Interferon-γ and Colorectal Cancer: An up-to date. J. Cancer.

[33] Yu B., Wu K., Wang X., Zhang J., Wang L., Jiang Y., Zhu X., Chen W., Yan M. (2018). Periostin secreted by cancer-associated fibroblasts promotes cancer stemness in head and neck cancer by activating protein tyrosine kinase 7. Cell Death Dis..

[34] González-González L., Alonso J. (2018). Periostin: A Matricellular Protein With Multiple Functions in Cancer Development and Progression. Front. Oncol..

[35] Yang T., Deng Z., Pan Z., Qian Y., Yao W., Wang J. (2020). Prognostic value of periostin in multiple solid cancers: A systematic review with meta-analysis. J. Cell. Physiol..

[36] Bao S., Ouyang G., Bai X., Huang Z., Ma C., Liu M., Shao R., Anderson R.M., Rich J.N., Wang X.-F. (2004). Periostin potently promotes metastatic growth of colon cancer by augmenting cell survival via the Akt/PKB pathway. Cancer Cell.

[37] Li Z., Zhang X., Yang Y., Yang S., Dong Z., Du L., Wang L., Wang C. (2015). Periostin expression and its prognostic value for colorectal cancer. Int. J. Mol. Sci..

[38] Oh H.J., Bae J.M., Wen X.-Y., Cho N.-Y., Kim J.H., Kang G.H. (2017). Overexpression of POSTN in Tumor Stroma Is a Poor Prognostic Indicator of Colorectal Cancer. J. Pathol. Transl. Med..

[39] Ma Y., He L., Zhao X., Li W., Lv X., Zhang X., Peng J., Yang L., Xu Q., Wang H. (2021). Protease activated receptor 2 signaling promotes self-renewal and metastasis in colorectal cancer through β-catenin and periostin. Cancer Lett..

[40] Huizer K., Zhu C., Chirifi I., Krist B., Zorgman D., van der Weiden M., van den Bosch T.P.P., Dumas J., Cheng C., Kros J.M. (2020). Periostin Is Expressed by Pericytes and Is Crucial for Angiogenesis in Glioma. J. Neuropathol. Exp. Neurol..

[41] Righi A., Sarotto I., Casorzo L., Cavalchini S., Frangipane E., Risio M. (2015). Tumour budding is associated with hypoxia at the advancing front of colorectal cancer. Histopathology.

[42] Qiu F., Shi C.-H., Zheng J., Liu Y.-B. (2013). Periostin mediates the increased pro-angiogenic activity of gastric cancer cells under hypoxic conditions. J. Biochem. Mol. Toxicol..

[43] Tung K.-H., Lin C.-W., Kuo C.-C., Li L.-T., Kuo Y.-H., Lin C.-W., Wu H.-C. (2013). CHC promotes tumor growth and angiogenesis through regulation of HIF-1α and VEGF signaling. Cancer Lett..

[44] Yang P., Yu P.B. (2020). Periostin: A Novel Integrator of Hypoxic Signaling in Pulmonary Hypertension. Circ. Res..

[45] Mirza R.E., Fang M.M., Novak M.L., Urao N., Sui A., Ennis W.J., Koh T.J. (2015). Macrophage PPARγ and impaired wound healing in type 2 diabetes. J. Pathol..

[46] Servais C., Erez N. (2013). From sentinel cells to inflammatory culprits: Cancer-associated fibroblasts in tumour-related inflammation. J. Pathol..

[47] Raz Y., Erez N. (2013). An inflammatory vicious cycle: Fibroblasts and immune cell recruitment in cancer. Exp. Cell Res..

[48] Kumar P., Smith T., Raeman R., Chopyk D.M., Brink H., Liu Y., Sulchek T., Anania F.A. (2018). Periostin promotes liver fibrogenesis by activating lysyl oxidase in hepatic stellate cells. J. Biol. Chem..

[49] Castro F., Cardoso A.P., Gonçalves R.M., Serre K., Oliveira M.J. (2018). Interferon-Gamma at the Crossroads of Tumor Immune Surveillance or Evasion. Front. Immunol..

[50] Dinarello C.A. (2018). Overview of the IL-1 family in innate inflammation and acquired immunity. Immunol. Rev..

[51] Corliss B.A., Azimi M.S., Munson J.M., Peirce S.M., Murfee W.L. (2016). Macrophages: An Inflammatory Link Between Angiogenesis and Lymphangiogenesis. Microcirculation.

[52] Hayashi N., Yoshimoto T., Izuhara K., Matsui K., Tanaka T., Nakanishi K. (2007). T helper 1 cells stimulated with ovalbumin and IL-18 induce airway hyperresponsiveness and lung fibrosis by IFN-gamma and IL-13 production. Proc. Natl. Acad. Sci. USA.

[53] Hus I., Maciag E., Roliński J. (2010). The role of Th17 cells in anti-cancer immunity. Postepy Hig. Med. Dosw. (Online).

[54] Chen Y., Tan W., Wang C. (2018). Tumor-associated macrophage-derived cytokines enhance cancer stem-like characteristics through epithelial-mesenchymal transition. OncoTargets Ther..

[55] Vilcek J., Palombella V.J., Henriksen-DeStefano D., Swenson C., Feinman R., Hirai M., Tsujimoto M. (1986). Fibroblast growth enhancing activity of tumor necrosis factor and its relationship to other polypeptide growth factors. J. Exp. Med..

[56] Zhu D., Zhou W., Wang Z., Wang Y., Liu M., Zhang G., Guo X., Kang X. (2021). Periostin: An Emerging Molecule With a Potential Role in Spinal Degenerative Diseases. Front. Med. (Lausanne).

[57] Blauvelt A., Chiricozzi A. (2018). The Immunologic Role of IL-17 in Psoriasis and Psoriatic Arthritis Pathogenesis. Clin. Rev. Allergy Immunol..

[58] Xu X., Chang W., Yuan J., Han X., Tan X., Ding Y., Luo Y., Cai H., Liu Y., Gao X. (2016). Periostin expression in intra-tumoral stromal cells is prognostic and predictive for colorectal carcinoma via creating a cancer-supportive niche. Oncotarget.

[59] Schürch C.M., Bhate S.S., Barlow G.L., Phillips D.J., Noti L., Zlobec I., Chu P., Black S., Demeter J., McIlwain D.R. (2020). Coordinated Cellular Neighborhoods Orchestrate Antitumoral Immunity at the Colorectal Cancer Invasive Front. Cell.

